# Adherence to complementary feeding guidelines among caregivers of children aged 6-23 months in Lamwo district, rural Uganda

**DOI:** 10.11604/pamj.2018.31.17.14955

**Published:** 2018-09-06

**Authors:** Harriet Aber, Angela Nakanwagi Kisakye, Juliet Ndimwibo Babirye

**Affiliations:** 1 Makerere University College of Health Sciences School of Public Health, Kampala, Uganda; 2 African Field Epidemiology Network, Kampala, Uganda

**Keywords:** Complementary feeding, breast feeding, adherence, Lamwo, attitudes, IYCF and malnutrition

## Abstract

**Introduction:**

Malnutrition contributes to half of all deaths among children under-five years in developing countries such as Uganda. Optimal complementary feeding is one of the crucial interventions that could prevent these deaths. This study measured adherence to complementary feeding guidelines and its associated factors among caregivers of children aged 6-23 months in Lamwo district, rural Uganda.

**Methods:**

A household cross-sectional study was used to collect data on adherence to complementary feeding among 349 caregivers. A composite variable with 9 indicators of complementary feeding was used to measure adherence. Univariable and multivariable logistic regression was used for statistical analysis using STATA software.

**Results:**

A household cross-sectional study was used to collect data on adherence to complementary feeding among 349 caregivers. A composite variable with 9 indicators of complementary feeding was used to measure adherence. Univariable and multivariable logistic regression was used for statistical analysis using STATA software.nearly all (97.7%, 341/349) children had ever been breastfed. Complementary feeding was initiated at six months for 47.0% (164/349) of the children. The number of complementary meals ranged from 1-4 meals per day with a mean of 3 meals per day (SD = 0.8). About half (55.8%, 195/349) of the children were given less than the recommended amount of food. Overall only 40.1% (140/349) of all study respondents were adherent to complementary feeding guidelines. The odds of adherence to complementary feeding were higher among caregivers with children aged 6-8 months (AOR = 4.68, 95% CI: 1.91-11.48), children whose fathers had attained 8 or more years of formal education (AOR = 2.27, 95% CI: 1.22-4.19), caregivers with two children under five years (AOR = 5.46, 95% CI: 1.46-20.36), those living in the poorest households (AOR = 3.00, 95% CI: 1.37-6.57) and those who showed willingness to recommend initiation of complementary feeding at six months to another mother (AOR = 1.34 95% CI: 1.06-1.70).

**Conclusion:**

Adherence to complementary feeding guidelines was very low in this rural African setting indicating an urgent need for interventions such as health education to improve adherence with consequent reduction in rates of under nutrition. These interventions should target caregivers with older children, fathers with less than 8 years of formal education and those living in the wealthiest households.

## Introduction

Globally child survival continues to be a priority since it was estimated in 2015 that 5.9 million children under five years died from preventable diseases [1]. Half of these deaths resulted from diseases related to under-nutrition. In Uganda, over 33% of children under-five years are stunted, a situation which is classified as a serious public health problem according to the World Health Organization (WHO) [2]. Adequate infant and young child feeding can improve child survival and development with evidence showing that applied breastfeeding and complementary feeding practices save the lives of more than three million children under-five years annually [3]. Nutritional interventions in the first two years of a child's life are particularly important, as adequate nutrition during this period leads to reduced morbidity, mortality and to overall better development. Under-nutrition at this early age also entraps societies in the “cycle of poverty” [1, 3, 4] with considerable financial loss to national economies of up to 3.5 trillion dollars per year globally and 25 million dollars every year in Sub-Saharan Africa alone [5]. Malnutrition is a complex phenomenon that stems from various underlying determinants, including a lack of optimal feeding practices for infants and young children [4, 6]. According to the “Ugandan policy document on infant and young child feeding of 2004”, complementary feeding should start at 6 months with continued breastfeeding until two years of age or beyond. In addition, complementary food should be of correct consistency and nutrient dense [7]. However, foods in the complementary feeding period are often introduced too late for most children or they receive less than the recommended meals per day [8, 9]. This low adherence to complementary feeding guidelines means that many children continue to be vulnerable to under-nutrition including irreversible outcomes of stunting, poor cognitive development and significantly increased risk of infectious diseases [1, 5]. Lamwo district was selected for this study because of its reportedly high level of stunting of 41%, which according to the WHO classification makes it a critical public health problem. According to a nutrition surveillance report 2011, Lamwo district reported the highest levels of severe acute malnutrition among five Northern districts in Uganda. In addition the district also reported the lowest prevalence for infant feeding indicators [10]. Interventions such as targeted supplementary feeding, deworming, Vitamin A supplementation and education on infant and young child feeding guidelines have been implemented however, high levels of malnutrition persist [6, 7]. It is unclear why this is so, thus we measured adherence to complementary feeding guidelines and its associated factors among caregivers of children aged 6-23 months in Lamwo district so as to inform implementation of the policy on Infant and Young Child Feeding.

## Methods

**Study design and setting:** We used a cross sectional study design to collect data on adherence to complementary feeding (IYCF) Guidelines among mothers and caregivers of children aged 6-23 months of age in Lamwo District in April 2015.

**Sample size and sampling procedure:** We estimated a sample size of 350 using the formula for cluster surveys by Bennett *et al* [11] with the following assumptions: prevalence of appropriate complementary feeding in Uganda 28% [12], precision of 0.03, design effect of 1.6, intra-cluster variability of 0.1 [11], estimated number of respondents in a cluster 7. We randomly sampled 50 villages out of a total of 358 villages in the district [13] and randomly selected seven households from each village using a sampling procedure adapted from the WHO reference manual for cluster surveys 2005. At the household level, we identified children aged 6-23 months using a child health card or from recall.

**Measurements:** We measured Adherence to complementary feeding using nine indicators for adequate complementary feeding as adapted from the field manual by UNICEF and WHO [14]. Complementary feeding was defined using the following indicators: correct Time of start, correct amount of food, dietary diversity, correct meal frequency, meal consistency, hygiene practices, responsive feeding and correct feeding during illness. All the nine variables under complementary feeding were scored as either 1 or 0 to indicate adequate or inadequate practice respectively. We generated a composite variable with a maximum score of 9; any participant that scored ≥6 was categorized as adherent to the guidelines and any participant that scored ≤ 5 was considered as non-adherent [15-17]. We measured attitude towards complementary feeding on an ordinal scale using the following attitudinal statements: 1) initiation of semi-solid and solid food should start at 6 months of age, 2) children should continue to breastfeed even upon starting to eat solid foods until above 2 years, 3) breast milk alone is no longer enough for a child after 6 months, 4) I am willing to give my child solid food at 6 months, 5) I can recommend feeding of solid food to a mother with a 6 months child. We created a dichotomous variable for each statement, with 1 assigned to those who agree and 0 to those that disagreed with the statement. We summed up the scores and categorized a maximum score of 3-4 as positive attitudes and those with ≤2 statements were considered as negative. We constructed a measure of subjective norms by summing the responses to five statements; “I fed my baby solid foods for the very first time because: “I fed my baby solid foods (i.e., anything besides breast milk, formula, or water) for the very first time because: 1) a doctor or health professional said my baby should begin eating solid foods, 2) my baby's other parent said our baby should begin eating solid foods, 3) relatives said my baby should begin eating solid foods, 4) friends said my baby should begin eating solid foods, 5) my employer said my baby should begin eating solid foods. Those who responded “strongly agree/agree” to ≥ 3 items we categorized as having high subjective norm influence and those that strongly disagreed/disagreed with ≤ 2 of the items we categorized as having low subjective norm influence. We measured perceived behavioural control by summing the responses to five statements namely: I fed my baby solid foods (i.e., anything besides breast milk, formula, or water) for the very first time because...1) My baby wanted the food I ate, 2) My baby seemed unsatisfied with breast milk/formula, 3) I wasn't producing enough breast milk, 4) How much control did you have over the decision to introduce your baby to solid foods? 5) How much control do you currently have over what you feed your baby on a daily basis? Respondents who strongly agreed/agreed to ≥ 3 items we categorized as having high control and those with ≤2 of the items as having little or no control. We developed a household wealth index by using principal components analysis with variables on asset ownership. Regression factor scores generated from the first principal component were ranked in ascending order and then categorized into quintiles (1) poorest, to (5) least poor.

**Data collection and management:** We collected data using a structured questionnaire that was translated into Luo (the local language commonly spoken in Lamwo). We trained five data collectors for two days on sampling and interviewing techniques prior to the survey.

**Data entry and analysis:** We entered data into excel spread sheets and analysed using STATA software version 12. Statistically significant response patterns were considered if a two-sided p-value was <0.05. We calculated means and proportions for socio demographic characteristics and feeding practices. All variables with a p-value < 0.2 at bivariable analysis were considered in multivariable analysis using the forward stepwise multivariable logistic regression model. We also used Adjusted odds ratios (AOR) and 95% Confidence intervals to identify independent predictors of adherence to feeding guidelines.

**Ethical consideration:** We obtained ethics approval for the study from the Makerere University School of Public Health Higher Degrees Research and Ethics committee. Study participants provided informed written consent before participation in the study.

## Results

**Socio demographic characteristics of caregivers and children in Lamwo district, April 2015:** The study had a 100% response rate with caregivers' ages ranging from 18-56 years, with a mean age of 27.1 years (±standard deviation, SD = 6.3). The household size ranged from two-fifteen people, with a mean size of five people (±2.0) and about 57.5% of households had ≥5 family members. More than half 218 (62%) of the children were >12 months of age with a mean age of 13.7 (±5.4) (Table 1).

**Table 1:** Socio-demographic characteristics of the respondents and their children in Lamwo district, April 2015

Variables	Frequency	Percentage
	n=349	
**Age of the caregiver (years)**		
< 19	31	8.9
20-29	199	57
> = 30	119	34.1
**Educational status of the caregiver**		
0-7 years	264	75.7
8-16 years	85	24.3
**Educational status of the father of study child**		
0-7 years	152	43.7
8-16 years	196	56.3
**Children under five years of age**		
1	170	48.9
2	150	43.1
3	28	8.1
**Caregiver is engaged in formal employment**		
No	203	58.2
Vendor	43	12.3
Farmer	80	22.9
Office worker	10	2.9
Others	13	3.7
**Marital status**		
Cohabiting	210	60.2
Married	114	32.7
Divorced/separated	20	5.7
Widow	5	1.4
**Wealth index**		
Q1Poorest	70	20.1
Q2 Poor	110	31.5
Q3 Middle	30	8.6
Q4 Rich	80	22.9
Q5 Richest	59	16.9
**Age of children**		
6-8 months	71	20.3
9-11 months	60	17.2
12-23 months	218	62.5

**Feeding practices:** Overall, adherence to complementary feeding guidelines was only 140 (40.1%). Nearly all 341(97.7%) of the children had ever breastfed and 318 (91.1%) received a syrup or capsule for vitamin A supplementation. The number of complementary meals in our study ranged from one-four meals per day with a mean of three meals (SD = 0.8). Less than half 148 (42.4%) of the children were reported to have received at least four of the foods from the seven recommended food groups (Table 2).

**Table 2:** Feeding practices of children aged 6-23 in Lamwo district, April 2015

Variables	Frequency	Percent
Time of initiation		
At 6 months	164	47
Before 6 months	30	8.6
After 6 months	155	44.4
**Continued breastfeeding at the time of the study**		
Yes	295	84.5
**Frequency of meals per day**		
Yes	224	64.2
**Correct amount of food taken per day**		
Yes	154	44.1
**Consistency of food**		
Appropriate	113	32.4
At least four food groups given		
Yes	148	42.4
**Hygienic principles**		
Yes	255	73.1
Responsive feeding		
Yes	304	87.1
**Feeding during illness**		
Adequate	135	38.7
**Adherence to complementary feeding**		
Yes	140	40.1
No	209	59.9

**Respondent characteristics and adherence to complementary feeding:** The odds of adherence to complementary feeding were higher among young child caretakers, those with children aged 6-8 months, caretakers or their spouses who had attained more than 8 years of formal education, those with one or two children under five years of age, those engaged in formal employment and those living in the poorest wealth quintile.

**Attitudes towards adherence to complementary feeding:** More than half 253 (72.4%) of the respondents had a positive attitude towards complementary feeding. At univariable analysis, a statistically significant association was observed between adherence to complementary feeding guidelines and caregivers' willingness to recommend feeding solid foods to children at six months (UOR = 2.03, 95% CI: 1.19-3.46) (Table 3).

**Table 3:** Multivariable analysis of factors associated with adherence to complementary feeding among caregivers of children aged 6-23 months in Lamwo district, April 2015

Variables	Adherent to CF	Unadjusted	Adjusted	p-value
	n=140	OR (95%CI)	OR (95% CI)	
**Age of the caregiver (years)**				
< 19	18(47.3)	2.36(1.05-5.27)	1.58(0.50-4.99)	0.435
20-29	78(39.2)	1.09(0.68-1.75)	0.85(0.40-1.81)	0.684
>=30	44(36.9)	1		
**Age of the study child (months)**				
6-8 months	42(59.2)	2.68(1.32-5.47)	4.68(1.91-11.48)	0.001
9-11 months	21(35.0)	1		
12-23 months	77(35.3)	1.01(0.55-1.84)	1.53(0.73-3.21)	0.253
**Educational status of the father of study child**				
0-7 years	36(23.6)	1		
8-16 years	104(53.0)	3.64(2.28-5.8)	2.27(1.22-4.19)	0.009
**Children under five years of age**				
1	67(39.1)	2.83(1.02-7.84)	3.52(0.88-14.02)	0.074
2	68(45.0)	3.60(1.29-10.02)	5.46(1.46-20.36)	0.011
3	5(18.5)	1		
**Wealth index**				
Bottom quintile, Poorest	42(60.0)	3.5(1.86-6.56)	3.00(1.37-6.57)	0.006
2^nd^quintile	33(30.0)	1		
3^rd^quintile	14(46.7)	2.04(0.89-4.65)	1.40(0.50-3.94)	0.519
4^th^quintile	26(32.5)	1.12(0.60-2.08)	1.19(0.57-2.48)	0.627
**Top quintile, Least poor**	25(42.4)	1.71(0.88-3.31)	1.79(0.81-3.96)	0.146
**I can recommend another mother feed their child solid food at 6 months**				
Yes	116(44.1)	2.03(1.19-3.46)	1.34(1.06-1.70)	0.012
No	24(27.9)	1		
**How much control do you currently have over what you feed your baby on a daily basis?**				
Yes	86(38.0)	1		
No	54(46.1)	1.45(0.92-2.28)	1.09(0.50-2.36)	0.816

**Influence of others on adherence to complementary feeding:** About a third 130 (37.3) of caregivers were highly influenced by others in their decisions. At univariable analysis, respondents that reported influence from the father of the child (UOR = 2.0, 95% CI: 1.26-3.11), relatives (UOR = 2.45, 95% CI: 1.52-3.48), friends (UOR = 2.18, 95% CI: 1.36-3.11), or local leaders (UOR = 1.67, 95% CI: 1.04-2.70) were more likely to adhere to complementary feeding guidelines (Table 3).

**Perceived behavioural control to complementary feeding:** Over half 201(57.5%) of the respondents had low perceived behavioural control. At bivariable analysis, the odds of adherence to complementary feeding were higher among caregivers who had high-perceived behavioural control (UOR= 1.94, 95% CI: 1.24-3.03) however, this association was not observed at the multivariable analysis (Table 3).

**Factors independently associated with adherence to complementary feeding guidelines:** The odds of adherence to complementary feeding guidelines were higher among caregivers with children aged 6-8 months (AOR = 4.68, 95% CI: 1.91-11.48), those with two children under five years of age (AOR = 5.46, 95% CI: 1.46-20.36), those whose spouses had attained more than 8 years of formal education (AOR = 2.27, 95% CI: 1.22-4.19), those living in the poorest households (AOR = 3.00, 95% CI: 1.37-6.57) and those who showed willingness to recommend initiation of complementary feeding at six months to another mother (AOR = 1.34 95% CI: 1.06-1.70) (**Table 3**).

## Discussion

This study measured adherence to complementary feeding guidelines and its associated factors. Overall, 40% of all respondents were adherent to the WHO guidelines on complementary feeding. Caregivers with children aged 6-8 months, those with two children under five years of age, those whose spouses had attained more than 8 years of formal education, those living in the poorest households, and those who showed willingness to recommend initiation of complementary feeding at six months to another mother had higher odds of adherence to complementary feeding guidelines. The level of adherence to complementary feeding guidelines in our study was higher than the national average of 6% reported in 2011 [6]. The Uganda infant and young child feeding guidelines adapted from the global WHO guidelines have been operational in Uganda since 2004 however, despite their enactment, infant feeding practices have remained poor with no improvement for complementary feeding practices particularly. This stagnancy can be observed in the 2006 Demographic and Health Survey (DHS) that reported only 24% of caregivers as having adhered to complementary feeding guideline [18]. Adherence to complementary feeding further deteriorated with estimates from the 2011 DHS showing that only 6% of the children were being fed correctly [6]. An earlier study indicated that although adherence to feeding guidelines was higher among HIV positive Ugandan mothers who had received infant feeding counselling, however, infant feeding counselling was rare in this setting [19]. A subsequent study showed continued low level of infant feeding counselling in this setting [20]. It is critical that effective interventions such as infant feeding counselling are rolled out across all Ugandan health facilities and communities in order to improve adherence to guidelines. In our study, the caregiver's age was not statistically associated with adherence to complementary feeding guidelines similar to findings from South India in a study done to establish complementary feeding practices among mothers of children aged 6months to 2 years [8]. In contrast, an Australian study that used a complementary feeding index found an association between older maternal age and adherence to complementary feeding [15]. The difference in findings could be due to the fact that Australian mothers were better educated than the Ugandan mothers. The highly prevalent lower education among our study participants may partly explain why we did not find a statistical difference in adherence to complementary feeding guidelines due to maternal level of education. In other settings, maternal education has been found to correspond to adherence to feeding guidelines and in these settings higher proportions of adherence to feeding guidelines are reported among mothers with more than 7 years of formal education [9, 21, 22]. Similarly, higher levels of adherence to complementary feeding guidelines have been reported among mothers with spouses that have attained more than 7 years of formal education comparable to our study findings. Another study suggested that education enables husbands to understand their wives and provide help and approval to what mothers would like to do to keep the child healthy [22].

In our study the child's age (6-8 months) was associated with higher odds of adherence to complementary feeding guidelines similar to findings from urban slums in India [23]. However, a cohort study in a Nigerian hospital found that complementary feeding was highest in the age group 9-11 months in comparison to other age categories [24]. This difference in findings might be due to the fact that the Nigerian study was conducted in a hospital essentially introducing selection bias unlike our study, which was conducted at the household level. Furthermore, having two children less than five years was a predictor for adherence to complementary feeding in this study similar to findings from a high-income setting [25]. Although it may be difficult to completely decipher the reasons for this observation, it is possible that mothers with more than one child have high levels of infant feeding related knowledge since they have past experience with infant feeding [26]. Living in the poorest households was associated with higher odds of adherence to complementary feeding guidelines contrasting findings from India where higher income was associated with increased adherence to infant feeding guidelines [17,22]. Besides the differences in the feeding cultures between the two study populations, poverty was highly prevalent among our study participants and most caregivers reported that they were unemployed. Previous child health research in Uganda shows that living in the poorest households is a predictor of non-adherence to child health guidelines which is a reflection of the prevailing health system failure that may not deliver information adequately to all populations [27,28]. Furthermore, it is expected that those living in the less poor households should adhere to feeding guidelines since they can better afford to purchase the requirements for adequate nutrition of the infants and young children. However, our study population were mainly unemployed which means that mothers spent more time caring for their children unlike mothers who were employed, this could explain why those in the poorest households were more likely adhere to feeding guidelines. The index generated in our study is unlike other complementary feeding indices such as the Complementary Feeding Utility Index (CFUI) which provides a summary score that encompasses nutritional, developmental, and behavioural complementary feeding guidelines [15]. Our findings were lower than other study reports that used composite variables in Australia and Burkina Faso [15,16]. However, those studies used fewer indices in their measurements. In interpreting the findings from our study, some limitations of the study design should be considered. First, a differential error in measurement could have arisen if the respondents that were not adherent to complementary feeding guidelines were less likely to report sub-optimal complementary feeding practices because of perceived unacceptability of this behaviour particularly consequent to previous advice on child feeding from health workers. Second, a study with a larger sample size would narrow the wide confidence intervals observed in our study. Lastly, we assessed feeding behaviour at one point in time. This overlooks the varying nature of feeding behaviour as the child grows, current feeding practice does not necessarily predict previous or future feeding patterns. The survey was conducted using the 2005 WHO methodology for cluster surveys which does not provide for probabilistic sampling. This means the proportions reported in this report tend to be higher than the actual expected measurements. Furthermore, the potential for selection bias was likely with the use of this 2005 WHO methodology for EPI surveys; arising from field practices such as starting enumeration from village centers, using household replacement, and including only current residents which misses mobile populations who may have worse feeding methods compared to those who had stayed in the area permanently [29, 30]. The bias could have been mitigated by use of a probability sample as suggested in the new 2015 WHO methodology for EPI cluster surveys. With the probability sampling method every eligible respondent in the cluster has a chance of being selected and weighted statistical analyses can be conducted [30].

## Conclusion

Adherence to complementary feeding guidelines was low (40%) in this rural African setting. The father's attainment of at least 8 years of formal education, having a child aged 6-8 months, belonging to the poorest wealth quintile and the willingness of the caregiver to recommend initiation of complementary feeding at six months to another mother were all predictive of adherence to complementary feeding. The use of a composite variable to measure adherence to complementary feeding provided a broad basis for understanding complementary feeding practices for rural African settings. Composite variables should be used regularly in the assessment of complementary feeding among programs. Additionally, in order to increase the level of adherence to guidelines, health educational interventions should focus on increasing understanding of the importance of adherence to complementary feeding guidelines particularly targeting fathers who are either illiterate or those of lower educational attainment, caretakers living in the less poor wealth quintiles and those with children older than 8 months of age. A large-scale longitudinal study using mixed methods should be designed to understand the persistent low adherence to complementary feeding guidelines in Uganda. In addition this study would show the variation in adherence to feeding guidelines for each individual child over time. The variation in the factors that affect adherence to complementary feeding over time would also be identified. Finally, further study of the influence of wealth status on feeding practices within a more heterogeneous population would further highlight the reasons for non-adherence to feeding guidelines.

### What is known about this topic

- Timely initiation of complementary feeding is low in Uganda and complementary feeding practices are poor;
- Factors associated with timeliness include, family size, education level of caregiver, number of children, age of child, etc.

### What this study adds

- This paper reports low adherence (40%) to complementary feeding guidelines in this rural African setting therefore the Ministry of Health should develop interventions to increase adherence to complementary feeding guidelines;
- Focus should be given to increasing the understanding of the importance of adherence to complementary feeding guidelines and targeting older children, fathers with less than 8 years of formal education and those living in the wealthiest households;
- Further research is also needed to observe, in a longitudinal study, the variation in adherence to guidelines for each child and to identify the varying nature of the factors that influence adherence over time.

## Competing interests

The authors declare no competing interest.
